# Housing conditions, satisfaction with housing conditions and life satisfaction of older adults in Tanzania: a cross-sectional study

**DOI:** 10.1186/s12877-026-07542-3

**Published:** 2026-04-28

**Authors:** Stellah John Kilawe, Huijun Liu, Zhibin Li

**Affiliations:** 1 Institute for Population and Development Studies, School of Public Policy and Administration, Xi’an Jiaotong University, Xi’an, China; 2 Institute of Rural Development Planning (IRDP), Dodoma, Tanzania

**Keywords:** Older adults, Housing conditions, Satisfaction with housing conditions, Life satisfaction, Heterogeneity

## Abstract

**Background:**

Housing conditions are a critical determinant of well-being among older adults. Yet, their influence on life satisfaction particularly the underlying mechanisms in developing countries, especially in Tanzania, remains underexplored.

**Objective:**

This study examines the impacts of housing conditions and satisfaction with housing conditions on life satisfaction among older adults in Tanzania. Specifically, it explores the associations between housing conditions, satisfaction with housing conditions, and life satisfaction, as well as the mediating role of satisfaction with housing conditions in the relationship between housing conditions and life satisfaction.

**Method:**

Data were derived from a cross-sectional survey conducted between January and March 2024. A multi-stage, stratified, probability-proportionate-to-size (PPS) sampling design was employed to recruit 2,012 older adults (aged 60 +) from households across four geographical zones/regions of Tanzania. Logistic regression was used to construct the baseline model, and the Karlson-Holm-Breen (KHB) decomposition method was applied to examine the associations among housing conditions, satisfaction with housing conditions, and life satisfaction.

**Results:**

Housing conditions were significantly and positively associated with life satisfaction (OR = 1.433, 95% CI: 1.186–1.731, *p* < 0.001). Satisfaction with housing conditions was also significantly and positively associated with life satisfaction (OR = 1.666, 95% CI: 1.591–1.744, *p* < 0.001). Furthermore, satisfaction with housing conditions mediated the association between housing conditions and life satisfaction, accounting for 37.4% of the relationship. The effect of housing conditions on life satisfaction varied significantly by geographical zone/region, whereas no significant differences were observed by gender or urban–rural residence.

**Conclusion:**

These findings suggest a need for more effective strategies and practical policies to improve housing conditions, thereby enhancing life satisfaction among older people in Tanzania. Additionally, regional planning and housing policy should prioritize addressing disparities in vulnerability and adversity faced by older adults in relation to housing.

**Supplementary Information:**

The online version contains supplementary material available at 10.1186/s12877-026-07542-3.

## Introduction

Tanzania’s older adult population is growing both in absolute numbers and as a proportion of the total population. The 2022 National Population Census indicates that 5.7% of the population was aged 60 years and older [15, 43–45], a proportion projected to double to 11% by 2050 [36]. This demographic shift is occurring in a context of significant vulnerability, where older adults face considerable hardships including violence, food insecurity, limited healthcare access, and persistent poverty [15, 18, 20]. Amid these challenges, housing defined as a social right encompassing safety, security, basic utilities, and a place for social connection [34, 40, 49] emerges as a uniquely important determinant of life satisfaction. A safe and secure home is a fundamental prerequisite for well-being in later life [2, 49], particularly because older adults spend most of their time at home [49]. However, in Tanzania’s resource-constrained setting, this basic need remains highly unmet [15, 39, 42, 45]. Crucially, the country is undergoing this rapid demographic transition without either a strong economic foundation or comprehensive welfare system, such as the kind that historically supported aging populations in developed nations [15, 39]. This gap suggests that housing quality may become a primary determinant of life satisfaction. Therefore, meeting the growing demand for adequate housing presents an urgent policy challenge, drawing research attention to the specific link between older adults’ housing conditions and their life satisfaction [13, 19, 32, 40].

This urgent challenge is underscored by Tanzania’s significant housing deficit and uneven development. The country is unlikely to achieve the Sustainable Development Goals (SDGs) target of ensuring access to adequate, safe, and affordable housing for all by 2030 [40]. Poor housing conditions are multifaceted, encompassing inadequate water sources, lack of improved sanitation, reliance on unclean energy, tenure insecurity, and poor structural quality [5, 17, 40]. A previous study estimated that approximately 5.62 million housing units are in poor condition in rural areas and 2.30 million in urban areas [40]. Although national data indicates progress, such as an increase in the use of modern construction materials from 46 to 79% of households between 2012 and 2018, severe urban–rural disparities persist in access to electricity and improved sanitation [28]. In response to these challenges and aligned with the WHO’s call for healthy aging and the SDGs, the Tanzanian government has implemented strategic initiatives, such as the revised 2017 framework for older adults’ social care covering health, economic, and housing needs [20, 44]. However, empirical evidence suggests that improvements in housing conditions have not kept pace with demographic growth and are not equitably distributed across all older adults [15, 18, 32]. Given that housing conditions and needs vary across different subgroups, such as by gender, location, and region [15, 18, 32], the benefits of improved housing on life satisfaction are likely heterogeneous. This context of uneven development and an incomplete policy response highlights the critical importance of investigating how objective housing conditions relate to the life satisfaction of Tanzania’s growing older adult population, and whether this relationship differs across key subgroups. Understanding this heterogeneity is vital for designing effective, targeted policies.

Housing need theory provides a foundational framework for understanding the relationship between objective housing conditions and life satisfaction [10]. This theory posits that adequate housing conditions are a fundamental prerequisite for overall life satisfaction, particularly in later life [26, 35]. This perspective is extended by housing deficit and psychological construct theories, which suggest that older adults cognitively evaluate their housing against personal and socio-cultural standards [26, 35]. Within this evaluative paradigm, satisfaction with housing conditions emerges from the perceived congruence between one's actual living environment and these internalized standards. Conversely, perceived incongruence generates residential dissatisfaction. The theory of residential satisfaction [1] further elaborates this as a dynamic, reciprocal process between the individual and their environment. Synthesizing these perspectives yields a coherent mediation pathway: objective housing conditions are theorized to influence life satisfaction indirectly, primarily through their impact on satisfaction with housing conditions. When conditions are perceived as deficient, this appraisal directly erodes housing satisfaction, which in turn diminishes overall life satisfaction. Despite its strong theoretical foundation, this integrated model lacks direct empirical validation, particularly among older adults in resource-constrained settings like Tanzania**.**

In recent years, factors associated with life satisfaction have received increased attention from empirical research [48], and empirical evidence suggests that life satisfaction is determined by sociodemographic characteristics such as health status, education level, financial sufficiency, and age [3, 8, 48]. Studies have also examined how housing-related elements impact overall life satisfaction, as it is widely believed that an individual’s housing conditions have a significant impact on life satisfaction. Existing research confirms a link between housing conditions and older adults’ life satisfaction [5, 41]. For example, research conducted in South Korea indicates that factors such as housing tenure, quality, and affordability significantly influence life satisfaction across age groups [47]. Previous studies have confirmed that housing conditions are positively associated with life satisfaction [34], with a particular focus on home ownership [14] and housing security. However, housing conditions as a complex, multifaceted concept [37, 47] have been measured in different ways by prior studies. A comprehensive measure of housing conditions may help achieve a better understanding of the association between housing conditions and life satisfaction. Despite this variability, housing conditions have consistently emerged as a significant predictor of life satisfaction [30, 48]. Nevertheless, the association between housing conditions and life satisfaction is not uniform; it exhibits considerable heterogeneity. For instance, in China, life satisfaction varies based on ownership type: sole ownership and property rights held by a spouse are associated with higher life satisfaction than ownership solely by oneself (husband or wife) [14]. Additionally, prior research indicates that males and females often exhibit different patterns of life satisfaction, suggesting that the impact of housing conditions on life satisfaction may vary by gender difference [11, 37]. Evidence shows that poor housing conditions are more pronounced in rural areas than in urban areas [46], which may significantly influence life satisfaction across these settings.

Satisfaction with housing conditions is a complex construct that has been used to predict life satisfaction and assess housing conditions. Satisfaction with housing conditions refers to the subjective state that residents develop towards their living environment, influenced by their evaluation of that environment [1]. Housing satisfaction has been specifically linked to housing, facilities, safety, and transport [2]. Studies have confirmed that satisfaction with housing conditions was significantly and positively associated with life satisfaction [25]. This relationship is commonly observed in developed countries. For instance, in South Korea, a study revealed that satisfaction with housing conditions was significantly associated with life satisfaction [31]. In emerging countries such as China, a study found that satisfaction with housing conditions was a crucial factor determining overall life satisfaction [48]. Notably, a recent Chinese study focusing on the aging population indicates that older adults who were satisfied with their housing conditions experienced an increase in life satisfaction [23].

Recent studies have begun examining the mechanisms underlying the association between housing conditions and life satisfaction. Among such studies, research from South Korea examined the possible mediating effect of self-esteem on the relationship between housing conditions and life satisfaction [21]. A recent study investigated how housing conditions (the vulnerability of single households compared to couple households) affect life satisfaction in older adult households, with depression as a mediating factor [16]. Another study from China, reported multiple mediation effects of control beliefs over the aging experience and self-esteem on the relationship between housing conditions and life satisfaction [23]. Additionally, a study found that social support and meaning in life played significant mediating roles in the association between living arrangements and life satisfaction [22]. However, no study examined the mediating effect of satisfaction with housing conditions on the association between housing conditions and life satisfaction.

In both Tanzania and the boarder Sub-Saharan Africa (SSA) region, a limited but growing literature has begun to explore this dynamic, though it often focuses on single dimensions like energy poverty [38]. Furthermore, while older adults’ satisfaction with specific life domains including housing has been documented [6], studies have not examined how domain-specific satisfaction, particularly with housing, impacts overall life satisfaction. Consequently, a significant gap remains: no study has comprehensively examined the effects of multidimensional housing conditions on life satisfaction, nor empirically tested the role of satisfaction with housing conditions as a potential mediator in this relationship among older adults in Tanzania.

Thus, based on the existing theoretical and empirical evidence, the current study aims to address these gaps by:(1) examining the impacts of housing conditions and satisfaction with housing conditions on life satisfaction; (2) investigating the regional, rural–urban, and gender differences in the impacts of housing conditions on life satisfaction; (3) investigating whether satisfaction with housing conditions mediates the association between housing conditions and life satisfaction among Tanzanian older adults.

### Data and methods

#### Data

Data for this cross-sectional study were collected between January and March 2024. A nationally representative sample of adults aged 60 years and above in Tanzania was selected using a multi-stage, stratified, probability-proportionate-to-size (PPS) sampling design was employed to select a nationally representative sample of adults aged 60 years and above in Tanzania.The sampling procedure was as follows: First, mainland Tanzania was stratified into six geographical zones. We selected four zones (Northern, Central, Coastal, and Southern Highlands) from these based on their high concentrations of older adults each of these four zones had a proportion of older adults exceeding the national average of 5.7% [28]. Second, seven region were randomly selected from these four zones, namely Kilimanjaro, Mtwara, Njombe, Dodoma, Pwani, Dar es Salaam, and Mbeya [28].

The planned sample size for each region was allocated proportionally to its share of the national older adult population, following a probability-proportionate-to-size (PPS) approach. The sampling then proceeded through three further stages: district, ward, and household selection. In the third stage selection (districts), two districts (or municipalities) were selected from each selected region with equal probability. Consequently, the planned regional sample size was divided equally between these two districts. For the fourth stage selection (wards), two wards were chosen within each selected district via simple random sampling to ensure balanced geographic representation. Lastly, for household and participant selection, households with at least one older adult were enumerated within each ward. A target number of households per ward was calculated to meet the district’s sample allocation, and households were selected using systematic random sampling. All older adults (aged 60 +) within the selected households were eligible and were invited to participate (see Supplementary Fig. 1 for recruitment flow).

Data were collected through face-to-face interviews conducted by trained enumerators using KoboCollect v2024.2.4 software on tablet or mobiles devices. The structured questionnaire was developed based on established theoretical frameworks and underwent expert review and cognitive pre-testing to ensure clarity and contextual relevance.

### Measurements

#### Dependent variables

Life satisfaction was measured by a single-item question. This item came from the World Values Survey Wave 7 for the years 2017–2021. Each participant was asked, “How satisfied or unsatisfied are you with your whole life?” Five responses were provided: very dissatisfied = 1, dissatisfied = 2, neutral = 3, satisfied = 4, very satisfied = 5, with higher scores indicating higher life satisfaction. Following the measure of many other studies on global life satisfaction [4, 29], the original measure of life satisfaction was then recoded into a dichotomous variable. The responses from 1 to 3 were classified as “Dissatisfaction”, and the responses for 4 and 5 then were classified as “Satisfaction”.

#### Independent variable

In this study, housing conditions refer to the objective conditions of participant’s housing. Following measures of housing conditions used in prior studies, housing ownership status, number of rooms, construction materials for roofing, wall and floor, type of toilets, source of energy for cooking and lighting, and source of water were concerned [24, 48]. In this study, eight questions were applied to measure housing conditions as presented in Table 1. Subsequently, we used Item Response Theory (IRT) to create a new variable representing latent traits in our dataset. IRT model techniques allowed us to analyse the relationship between the eight items that were used to measure housing conditions and their underlying latent traits by applying the 1-parameter logistic (1PL) model to estimate item parameters and individual trait scores.

**Table 1:** Housing conditions index

Items	Coding
Wall material	1 point if present concrete slab, sand/cement blocks, stones, baked/burnt bricks; 0 if poles and mud/mud and stones, mud bricks, mud only
Floor material	1 point if present cement, tiles; 0 if the earth
Roofing material	1 point if present iron sheets, asbestos sheet; 0 if mud grasses/bamboo, mud, and grass
The main source of water	1 point if present tape water within the household; 0 otherwise
Ventilation of the kitchen when cooking at home	1 point if the presence of kitchen ventilation, fan, or by opening windows; 0 if no ventilation
The main source of lighting	1 point if present electricity, solar energy; 0 if used kerosene lamp, coal, etc
Number of rooms in the household	1 point if present of > or = 3 room; 0 if < than 3 rooms
Ownership of the residential house	1 point if the House owner; 0 if otherwise

#### Mediator variable

The measure of satisfaction with housing conditions as a mediator focuses on four different dimensions of the residential situation, which are aligned with prior frameworks and studies [1, 31]. Four items were used for the measure as follows: overall satisfaction with the housing you currently live in “(House),” satisfaction with healthcare services “(Facilities),” satisfaction with transportation “(Transport), and” satisfaction with the protection “(Safety)”. A five-point Likert response was provided: very dissatisfied = 1, dissatisfied = 2, neutral = 3, satisfied = 4, very satisfied = 5, with higher scores indicating higher housing satisfaction. A composite variable of satisfaction with housing conditions was generated by aggregating responses from the four items. Higher scores indicate greater satisfaction with housing conditions.

#### Control variables

The following variables were included as covariates in the study: location (0 = urban, 1 = rural), region (1 = northern, 2 = central, 3 = southern, 4 = coastal), gender (0 = male, 1 = female), living period (a continuous variable), age (a continuous variable), marital status (1 = married, 0 = single), children status (0 = having children, 1 = without children), retirement status (1 = retired, 0 = not retired), pension status (1 = with pension, 0 = without pension), financial sufficiency status (0 = financial adequacy, 1 = not adequacy), membership/association status (0 = with membership 1 = without membership), chronic disease status (1 = presence of chronic disease, 0 = without chronic disease), preferred living arrangement (0 = otherwise, 1 = co-residences with children), disability status (1 = with disability, 0 = without disability), health insurance status (0 = Uninsured, 1 = insured).

#### Statistical analysis

We began by conducting a descriptive analysis using a chi-squared test and t-test to compare sociodemographic characteristics between older adults who were satisfied and dissatisfied with life. Next, we used logistic regression to examine the associations between housing conditions, satisfaction with housing conditions, and life satisfaction, after adjusting for covariates. Subsequently, we employed Fisher’s permutation test to assess rural–urban, gender, and regional differences in the associations among housing conditions, satisfaction with housing conditions, and life satisfaction. Finally, to investigate the mediating effect of satisfaction with housing conditions on the association between housing conditions and life satisfaction among Tanzanian older adults, we conducted the Karlson-Holm-Breen (KHB) decomposition method with a 5000 bootstrap simples method. Furthermore, we conducted sensitivity analysis for different dimensions of satisfaction with housing conditions (Supplementary Table 1). Additionally, the effect of the indirect path was computed. All analyses were performed using Stata 18 software.

## Results

### Descriptive analysis

Table 2 presents descriptive statistics for life satisfaction among 2,012 older adults in Tanzania. The mean age of respondents was 73.2 years (SD = 20.6). The sample was predominantly female (63.6%, n = 1,280), and the majority of participants resided in rural areas (61.1%, n = 1,229). With regard to marital status, 55.1% were married, and 44.9% were single (including widowed, divorced, or never married). The vast majority (95.1%) had children. In terms of socioeconomic status, 65.5% reported financial inadequacy, 62.4% were not retired, and 89.9% had no pension. Regarding health status, most participants (64.4%) reported having at least one chronic disease, while 15.9% had a physical disability. Overall, 53.7% (n = 1,081) reported being satisfied with life, while 46.3% (n = 931) reported dissatisfaction with life. Bivariate analysis revealed several factors significantly associated with life satisfaction. Geographic and residential characteristics showed strong associations. Significant regional differences in life satisfaction among older adults in Tanzania were evident (χ^2^ = 88.5, *p* < 0.001). Additionally, older adults who had lived longer in their residences reported higher life satisfaction levels than those who had lived there for a shorter period (χ^2^ = 133.2, *p* < 0.001). Age and gender differences were found between older adults satisfied with life and those dissatisfied, with females being more likely to report dissatisfaction than males (χ2 = 24.5, *p* < 0.001). Furthermore, older adults with chronic diseases were more likely to be dissatisfied with life compared to those without (χ^2^ = 5.3, *p* = 0.021). Similarly, disability was also a strong predictor of life satisfaction (χ^2^ = 16.3, *p* < 0.001), with disabled older adults reporting lower satisfaction. Approximately 61% of older adults who preferred to live with children were more likely to report dissatisfaction with their lives, compared with 39% who preferred other arrangements (χ^2^ = 33.7, *p* < 0.001). Additionally, about 65.5% of older adults who reported financial inadequacy (χ^2^ = 93.1,* p* < 0.001) were more likely to report dissatisfaction with life than those with financial adequacy.

**Table 2:** Sample characteristics and univariate analysis with status of life satisfaction among older adults in Tanzania

Variables	**Total (N = 2012)** **N (%)**	**Dissatisfaction** **(N = 931)** **N (%)**	**Satisfaction** **(N = 1,081) N(%)**	**F/χ**^**2**^	***P-value***
Location				0.1	0.904
Urban	783 (38.9)	361 (38.8)	422 (39.0)		
Rural	1,229(61.1)	570 (61.2)	659 (61.0)		
Region				88.5	< 0.001
Northern	487 (24.2)	247 (26.5)	240 (22.2)		
Central	263 (13.1)	174 (18.7)	89 (8.2)		
Southern	460 (22.9)	143 (15.4)	317 (29.3)		
Coastal	802 (39.9)	367 (39.4)	435 (40.2)		
Living period, M(SD)	50.03(53)	48.02(78.6)	51.8 (71.5)	133.2	< 0.001
Age, *M(SD)*	73.2 (20.6)	72.5 (30.4)	73.7 (27.8)	95.5	< 0.001
Gender				24.5	< 0.001
Male	732 (36.5)	392 (42.1)	340 (31.5)		
Female	1,280(63.6)	539 (57.9)	741 (68.5)		
Marital status				2.1	0.143
Single	904 (44.9)	402 (43.2)	502 (46.4)		
Marriage	1,108(55.1)	529 (56.8)	579 (53.6)		
Children status				1.6	0.201
Having children	1,913(95.1)	879 (94.4)	1,034(95.7)		
Not having children	99 (4.9)	52 (5.6)	47 (4.3)		
Preferred living arrangement				33.7	< 0.001
Otherwise	788 (39.2)	428 (46.0)	360(33.3)		
Co-residences with children	1,224(60.8)	503(54.0)	721(66.7)		
Retirement status				2.5	0.113
Not retired	1,256(62.4)	564 (60.6)	692(64.0)		
Retired	756 (37.6)	367(39.4)	389(36.0)		
Pension status				1.4	0.239
Without pension	1,809(89.9)	845(90.8)	964 (89.2)		
With pension	203(10.1)	86 (9.2)	117(10.8)		
Membership/association status				0.5	0.502
With membership	288 (14.3)	128(13.7)	160(14.8)		
Without membership	1,724(85.7)	803(86.3)	921(85.2)		
Financial sufficient				93.1	< 0.001
Financial adequacy	695(34.5)	219(23.5)	476 (44)		
Financial inadequacy	1,317(65.5)	712(76.5)	605 (56)		
Health insurances status				3.7	0.053
Uninsured	1,293(64.3)	619(66.5)	674(62.3)		
Insured	719(35.7)	312(33.5)	407(37.7)		
Chronic diseases				5.5	0.021
Without chronic disease	717(35.6)	307(33)	410(37.9)		
Presence of chronic disease	1,295(64.4)	624(67)	671(62.1)		
Physical disabilities status				16.3	< 0.001
without disability	1,692(84.1)	750(80.6)	942(87.1)		
With Disability	320(15.9)	181(19.4)	139(12.9)		

*Note: * M = Mean; SD = Standard deviation; Rural residence was measured using administrative classifications based on Tanzania’s official ward designations. Interviewers recorded whether a participant’s location fell within a rural or urban ward, in accordance with government definitions;Disability indicates self-reported physical disability status ("Are you having any physical disabilities?" Yes/No); Membership/association status indicates participation in any formal or informal social groups (economic groups, neighborhood groups, religious groups, sports groups, or networking groups)

### Associations between housing conditions, satisfaction with housing conditions, and life satisfaction

The regression results investigating the impact of housing conditions and satisfaction with housing conditions on life satisfaction are presented in Table 3. Model 1 examined the association between housing conditions, satisfaction with housing conditions, and life satisfaction without adjusting for confounding variables. Results indicated a positive and significant association between housing conditions and life satisfaction (OR = 1.193**, 95% CI: 1.024–1.391), suggesting that older adults with good housing conditions were more likely to report satisfaction with life. Results in Model 1 also revealed that there is a strong positive association between satisfaction with housing conditions and life satisfaction (OR = 1.691***, 95% CI: 1.616–1.768). In Model 2, we included control variables. Notably, the association between housing conditions and life satisfaction not only remained significant (OR = 1.433***, 95% CI: 1.186–1.731) but strengthened, with the odds ratio increasing from 1.193 to 1.433. Similarly, satisfaction with housing conditions remained positively associated with life satisfaction (OR = 1.666***, 95% CI: 1.591–1.744). Among sociodemographic variables, region, gender, preferred living arrangement, retired status, and financial sufficiency emerged as significant predictors of life satisfaction.

**Table 3:** The associations between housing conditions, satisfaction with housing conditions, and life satisfaction

Predictor variables	Model 1	Model 2
	OR(CI)	OR(CI)
Housing conditions	1.193** (1.024–1.391)	1.433*** (1.186—1.731)
Satisfaction with housing conditions	1.691*** (1.616—1.768)	1.666*** (1.591—1.744)
Location (Ref. Urban)		1.119 (0.816—1.534)
Region (Ref. Northern)		
Central		0.253*** (0.153—0.416)
Southern		0.868 (0.535—1.408)
Coastal		0.908 (0.619—1.332)
Gender (Ref. Male)		1.448** (1.067—1.965)
Living period (continuous variable)		0.999 (0.993—1.006)
Age (continuous variable)		1.005 (0.989—1.020)
Marital status (Ref. Marriage)		1.011 (0.750—1.364)
Children status (Ref. Having children)		1.017 (0.587—1.763)
Preferred living arrangement (Ref. Otherwise)		1.616*** (1.194—2.186)
Retired status (Ref. Not retired)		0.690** (0.493—0.964)
Pension status (Ref. Without pension)		1.408 (0.842—2.353)
Membership/association status (Ref. With membership)		0.851 (0.575—1.259)
Financial sufficient status (Ref. Financial adequancy)		0.512*** (0.385—0.681)
Health insurance status (Ref. Uninsured)		1.084 (0.813—1.444)
Chronic disease status (Ref. Without chronic disease)		0.848 (0.627—1.147)
Disability status (Ref. without disability)		0.939 (0.655—1.347)
*Pseudo R*^*2*^* 0.4191*		*0.4545*
*N 2,012*		*2,012*

*Note: OR* Odd Ratio, *CI* Confidence Interval

Ref. = Reference group; N= Number of observations; Significance level: *p < 0.05,**p < 0.01,***p < 0.001

### Heterogeneity analysis

Table 4 presents the results of stratified analysis of regression analysis examining heterogeneity in the associations between housing conditions and life satisfaction with controlled covariates, across male–female, rural–urban, and across different region/zones. Findings reveal that housing conditions are significantly associated with life satisfaction among older adults in urban areas (OR = 1.467**, CI = 1.057–2.036) and those in rural areas (OR = 1.267*, CI = 0.992–1.620). Additionally, findings indicate that housing conditions are significantly associated with life satisfaction among male older adults (OR = 1.673***, CI = 1.213–2.309) and female older adults (OR = 1.284***, CI = 1.010–1.631).

**Table 4:** Regression results for life satisfaction by current living location, region and gender

Current living location			Region/zones				Gender	
Variables	Rural	Urban	Northern	Central	Southern highland	Coastal	Male	Female
Housing conditions	1.267*	1.467**	1.196	2.100*	1.638	1.242	1.673***	1.284***
	(0.992–1.620)	(1.057–2.036)	(0.859–1.666)	(1.186–3.721)	(0. 763–3.517)	(1.001–1.805)	(1.213–2.309)	(1.010–1.631)
Control variables Controlled								
Pseudo R^2^	0.517	0.388	0.348	0.402	0.719	0.454	0.449	0.457
N	1,229	782	486	263	460	802	732	1,280
**Fisher’s Permutation testfor group difference**								
Urban versus Rural		0.239						
Male versus Female		0.052						
North versus Central		0.036						
Central versus Southern highland		0.343						
Central versus Coastal		0.061						

*Note:* Significance level: *p < 0.05,**p < 0.01,***p < 0.001

A fisher permutation test was conducted to provide rigorous statistical evidence regarding significant heterogeneity (Table 4). The findings reveal that there is no significant difference in the effect of housing conditions on life satisfaction between rural and urban groups (*p* = 0.239). This suggests that housing conditions exert a similar impact on life satisfaction among older adults in both rural and urban areas. However, when examining regional differences in the association between housing conditions and life satisfaction, we found that housing conditions are significantly associated with life satisfaction only among older adults in the Central region (*p* = 0.038). Significant differences in the association were found between the Northern and Central regions, whereas there were no significant differences in the effect of housing conditions on life satisfaction between the Southern Highlands, Coastal, and Central regions. The findings also reveal that gender differences in the effect of housing conditions on life satisfaction is not statistically significant (*p* = 0.052).

### Mediation analysis

Table 5 presents the mediating effect of satisfaction with housing conditions on the association between housing conditions and life satisfaction using KHB Method with the 5000 bootstraps.

**Table 5:** Decomposition analysis effects between Housing Conditions and Satisfaction with Housing Conditions on Life Satisfaction using the KHB Method

Effects	Coefficient	P	Boot SE	Boot LLCI	Boot ULCI	Effects ratio
Indirect effect (a × b)	0.215	0.001	0.067	0.082	0.345	37.4%
Total effect (c)	0.575	0.001	0.117	0.335	0.794	
Direct effect(c')	0.360	0.001	0.095	0.170	0.545	

*Note: Boot* Bootstap, *SE* standard error, *LLCI* lower limit of the 95% Confidence interval, *ULCI* Upper limit of the 95% Confidence interval

Significance level: *p < 0.05,**p < 0.01,***p < 0.001

**Fig. 1:**
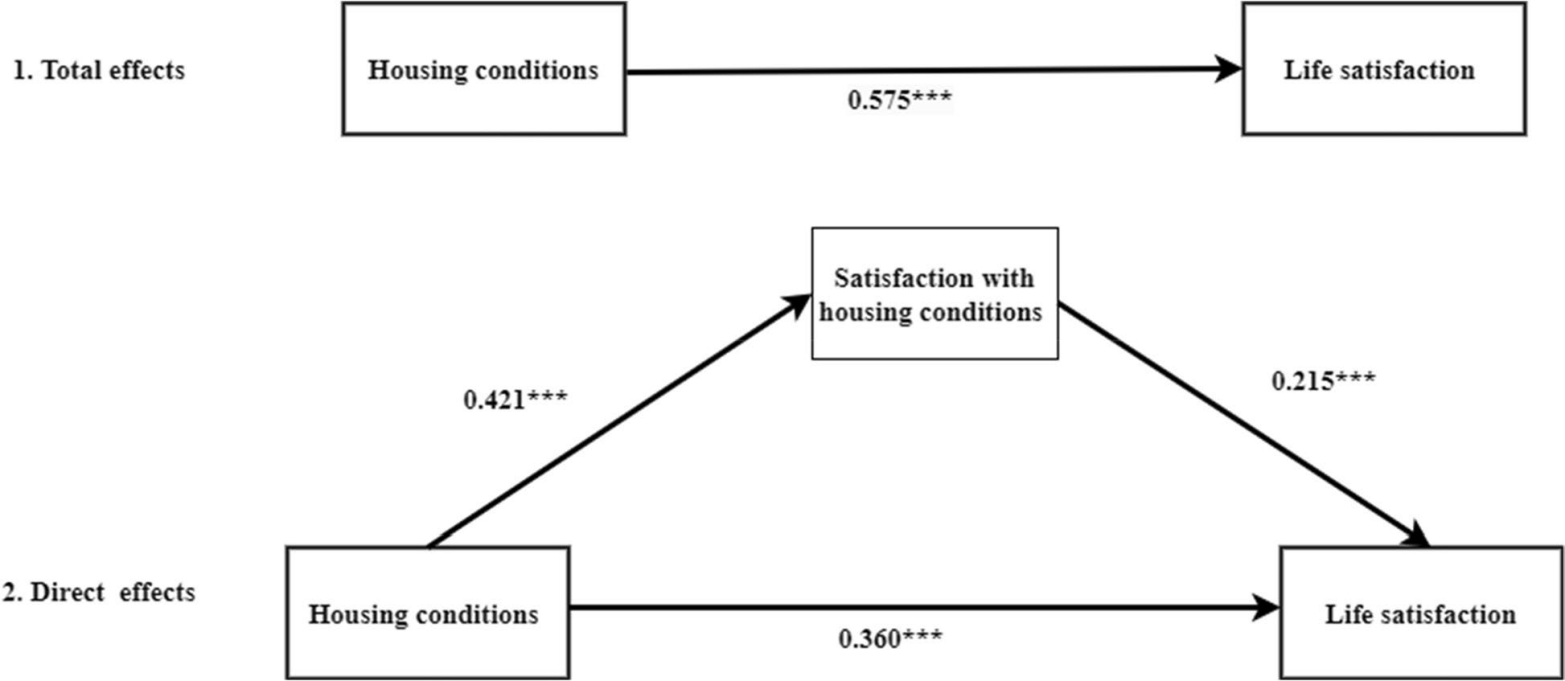
The association between housing conditions and life satisfaction as well the mediating role of satisfaction with housing conditions

The indirect effect of housing conditions on life satisfaction through satisfaction with housing conditions was significant (coefficient = 0.215, 95% CI = 0.087–0.351; *p* < 0.001), which suggests that housing conditions positively affect life satisfaction through satisfaction with housing conditions.The total effect (coefficient = 0.575, 95% CI = 0.273–0.728, *p* < 0.001) and direct effect (coefficient = 0.360, 95% CI = 0.170–0.545, *p* < 0.001) of housing conditions on life satisfaction were both significant. These results suggest that good housing conditions not only improve older adults life satisfaction directly but also enhance life satisfaction through positive perceptions of the housing conditions. Figure 1 illustrates the full mediation model, including the path from housing conditions to satisfaction with housing conditions (coefficient = 0.421, 95% CI = 0.166, 0.677, *p* < 0.001). Satisfaction with housing conditions exerts a partial mediating effect in the relationship between housing conditions and life satisfaction, and the indirect effect through satisfaction with housing conditions accounts for 37.4% of the total effect in the model.

## Discussion

To the best of our knowledge, this is the first study to examine the association between housing conditions measured as a comprehensive index and life satisfaction, as well as the mediating effect of satisfaction with housing conditions on the association between housing conditions and life satisfaction among older adults in Tanzania. Our analysis yielded four key findings. First, we found a strong link between better objective housing conditions and higher life satisfaction. Second, higher satisfaction with housing conditions was associated with higher life satisfaction among older adults. Third, and crucially, our analysis showed that satisfaction with housing conditions partially mediates the association between housing conditions and life satisfaction among older adults. Fourth, the analysis reveals significant regional heterogeneity in how housing conditions relate to life satisfaction. This regional variation deepens our understanding of the complex dynamics between housing and well-being in later life. Importantly, these findings are embedded within Tanzania’s distinct social and cultural context, where norms of intergenerational co-residence, the social value of home ownership, and community-based support systems profoundly shape housing expectations and experiences. For example, the weaker association observed in some regions may reflect variations in these local norms or in the implementation of national housing policies. For policymakers in Tanzania and similar contexts in Sub-Saharan Africa (SSA), a dual strategy is required: (1) continued investment in comprehensive improvement of objective housing conditions through national initiatives like the 2017 social care framework and the Tanzania National Human Settlements Development Policy), and (2) the adoption of participatory, culturally informed approaches that empower older adults. Such approaches directly address subjective satisfaction by aligning improvements with local expectations, thereby strengthening the pathway from housing to well-being. This integrated approach ensures that housing interventions are both materially sound and psychologically meaningful, maximizing their impact on quality of life for older adults. Importantly, satisfaction with housing conditions can also be enhanced through policies targeting healthcare accessibility, transportation, and safety for example, by expanding mobile healthcare visits for older adults, introducing age-friendly transport vouchers, and strengthening community policing or village council-based tenure recognition all of which are feasible within existing resource constraints in Tanzania.

In this study, the prevalence of life satisfaction and dissatisfaction among older adults in Tanzania was 53.7% and 46.3%, respectively. Significant differences were observed in the sociodemographic characteristics of those who were satisfied versus those who were dissatisfied, consistent with prior research [15, 39]. Our analysis revealed that a preference for living arrangements other than co-residence with children was associated with lower life satisfaction. This aligns with other studies [7] and suggests that for many older adults, this preference may signal an unmet need or a gap between desired and actual living situations factors potentially linked to lower social support and connection. Furthermore, supporting conclusions from prior studies [3, 12], we also found that financial adequacy and not being retired were independently associated with higher life satisfaction.

Although previous studies have well-documented the positive association between housing conditions and life satisfaction [47, 48], this study contributes to the literature by providing more comprehensive evidence on the impact of housing conditions on life satisfaction among older adults in Tanzania. Our findings indicate that the comprehensive index of housing conditions including wall material, floor material, roofing material, main source of water, ventilation of the kitchen when cooking at home, main source of lighting, number of rooms in the household, and home ownership was significantly associated with life satisfaction among older adults in Tanzania. Unlike earlier research, which has often examined only one or a few housing aspects in isolation, our approach offers a more holistic view. For example, prior studies have focused on specific factors such as home ownership [14], positive tenant experiences, property quality, and affordability [34]. By incorporating multiple dimensions of housing conditions, this study provides a more nuanced understanding of their effects on older adults' life satisfaction. Furthermore, these results offer empirical confirmation of the central proposition of housing need theory, which posits that adequate housing conditions are a fundamental prerequisite for overall life satisfaction, particularly in later life [26, 35].

Furthermore, this study provides a heterogeneity analysis of the relationship between housing conditions and life satisfaction among older adults in Tanzania. However, in contrast to some prior studies [33], we found no significant difference in this relationship between rural and urban areas: housing conditions were a significant predictor of life satisfaction in both contexts. In contrast with other research [37], our analysis revealed no gender difference in this association. Crucially, however, we contribute to the literature by demonstrating significant heterogeneity across Tanzania’s geographic regions. The impact of housing conditions on older adults’ life satisfaction was statistically significant only in the Central region and not in the Northern, Coastal, or Southern Highlands regions. This finding indicates that the importance of housing conditions for life satisfaction is not uniform but is instead highly context-dependent. In the Central region, housing quality may be a more salient and immediate determinant of life satisfaction, potentially because other supportive factors (e.g., social networks, economic security) are less available or stable, making the home environment disproportionately important. Conversely, in other regions, non–housing factors likely play a larger relative role. This heterogeneity underscores the need for regionally differentiated, rather than uniform national, policy approaches to improving life satisfaction among older adults in Tanzania.

In line with prior studies [23], this study confirms a strong positive association between satisfaction with housing conditions and life satisfaction among older adults in Tanzania. This finding provides new empirical support for housing need theory [26, 35] and validates the observation that older adults who perceive their housing conditions as adequate report higher levels of life satisfaction, while perceptions of unmet needs correlate with lower life satisfaction. While research in Tanzania has explored broad patterns of life satisfaction [6], this study provides the first specific evidence linking the subjective housing evaluation to overall well-being in this context. By doing so, our findings substantiate the theoretical model locally and suggest its broader utility for understanding older adults’ life satisfaction in similar socioeconomic and cultural settings across SSA. Consequently, this research offers a validated framework for investigating and addressing housing-related determinants of well-being in comparable SSA contexts.

This study confirmed satisfaction with housing conditions as a mediator in the association between housing conditions and life satisfaction among older adults in Tanzania. The study found that satisfaction with housing conditions mediates approximately 37.4% of the total effect of objective housing conditions on life satisfaction, which provides robust empirical validation for the integrated theoretical pathway proposed in this study. This substantial proportion indicates that the influence of physical housing quality on older adults' overall life satisfaction is not merely direct; rather, a significant portion operates through their subjective cognitive appraisal of those conditions. This result powerfully confirms the core proposition of housing deficit and psychological construct theories [9, 27]. The finding indicates that the influence of physical housing quality on an older adult’s overall life satisfaction is not merely direct; rather, a significant portion operates through their subjective cognitive appraisal. This finding demonstrates that older adults do not passively experience their housing, but actively evaluate it against their internalized standards, expectations, and needs. The 37.4% mediation effect quantifies the critical role of this evaluative process: when housing conditions are perceived as congruent with needs and expectations (high satisfaction), they strongly promote life satisfaction. When conditions are perceived as deficient (low satisfaction), they substantially diminish life satisfaction. Furthermore, this mediated pathway offers a crucial lens for policy and intervention in Tanzania and similar SSA contexts. Our findings suggest that improving life satisfaction requires more than just upgrading physical infrastructure; interventions must also address the subjective experience of housing by considering factors such as cultural appropriateness, perceived safety, and personal autonomy, which shape whether improved conditions are actually perceived as satisfactory. By identifying and quantifying this psychological bridge, our study moves beyond documenting a simple correlation to explaining how housing matters for life satisfaction among older adults, providing a more nuanced and actionable framework for promoting healthy aging in place.

This study has several limitations that should be acknowledged. First, housing conditions are a complex, multifaceted concept [37, 47]. Although we measured them using eight items, including ownership status, number of rooms, construction materials, energy sources, and water supply, other important dimensions, such as environmental health and safety hazards, were not captured. Future research should incorporate these additional aspects to provide a more comprehensive assessment of housing quality. Second, the analysis relied on cross-sectional data, which restricts our ability to establish temporal sequence or draw definitive causal claims. While this study examined the association between housing conditions and life satisfaction, along with the mediating role of satisfaction with housing conditions, longitudinal or experimental designs would be necessary to confirm the directionality and causality of these relationships. Third, for analytical purposes, we combined widowed, divorced, and never-married individuals into a single ‘unpartnered’ category due to limited subsample sizes. This aggregation may mask nuanced differences in their social support networks, economic resources, and psychological adjustment, all of which could independently influence well-being. Fourth, life satisfaction was measured using a single-item indicator from Wave 7 of the World Values Survey. While this measure has been widely used and accepted in previous cross-national aging research [31], and was chosen to maintain consistency with the survey protocol, we acknowledge that life satisfaction is a multidimensional construct. Single-item measures may fail to capture its full complexity. Future studies should consider employing multi-item scales such as Diener's Satisfaction with Life Scale to provide a more comprehensive assessment. Fifth, the focus on satisfaction with housing conditions as a mediator likely does not capture the full spectrum of mechanisms linking housing to well-being. Future studies should explore additional pathways through which housing conditions may influence life satisfaction, such as effects on physical health, social interaction opportunities, or feelings of security.

## Conclusion

Despite these limitations, this study represents an initial effort using cross-sectional data to demonstrate that housing conditions are associated with life satisfaction among older adults in Tanzania. Importantly, the effect of housing conditions on life satisfaction is mediated by satisfaction with housing conditions. As Tanzania experiences rapid population aging, satisfying older adults’ growing need for high-quality housing conditions has become a significant policy issue. The study found that housing conditions did not have a heterogeneous effect on life satisfaction between rural and urban areas or between older men and women. However, housing conditions exhibited significant regional variations in their effects on life satisfaction. Based on these findings, the National Human Settlements Development Policy should prioritize the development of comprehensive intervention strategies that target both multidimensional housing conditions and older adults’ satisfaction with these conditions. Such integrated approaches are essential to maintain and improve life satisfaction among older adults in Tanzania. To improve satisfaction with housing conditions without relying solely on physical housing upgrades, policymakers can implement low-cost measures such as mobile healthcare visits for older adults, age-friendly transport vouchers, and village council-based recognition of housing tenure to reduce safety and eviction concerns. Furthermore, more attention should be given to empowering regional social welfare departments in collaboration with regional advisory councils of elders. Providing these bodies with the necessary resources to function effectively will help address the disproportionate housing vulnerabilities and difficulties faced by older adults.

## Supplementary Information

Supplementary Material 1.

## Data Availability

Data will be made available to any researcher who agrees to comply with the guidelines set forth by the National Institute for Medical Research of Tanzania, as specified in the established research protocols.
