# Supplementary material for: Housing conditions, satisfaction with housing conditions and life satisfaction of older adults in Tanzania: a cross-sectional study
Source: BMC Geriatr. 2026 Apr 28;26:646. doi: 10.1186/s12877-026-07542-3 (PMC13154430; doi:10.1186/s12877-026-07542-3)
Supplement: Supplementary file 1 — Supplementary Material 1. [file 12877_2026_7542_MOESM1_ESM.docx]

Supplementary materials

Supplementary Table 1: Sensitivity Analysis of mediation effects of specific satisfaction with housing condition dimensions

| Satisfaction Dimension | Total Effect (c) | Direct Effect (c') | Indirect (Mediating) Effect (a*b) | Proportion Mediated |
| --- | --- | --- | --- | --- |
| 1. Housing (Dwelling) | 0.537*** | 0.195** | 0.342*** | 63.7% |
| 2. Facilities (Healthcare) | 0.551*** | 0.444*** | 0.107** | 19.4% |
| 3. Transportation | 0.512*** | 0.430*** | 0.081* | 15.9% |
| 4. Safety | 0.561*** | 0.517*** | 0.044* | 7.8% |

Note: Significance levels: ***p < 0.001, *p < 0.01, p < 0.05. Total Effect (c) = effect of housing conditions on life satisfaction without the mediator. Direct Effect (c') = effect of housing conditions on life satisfaction after including the mediator. Indirect Effect (a*b) = effect transmitted through the specified satisfaction dimension. Proportion Mediated = (Indirect Effect / Total Effect) × 100.


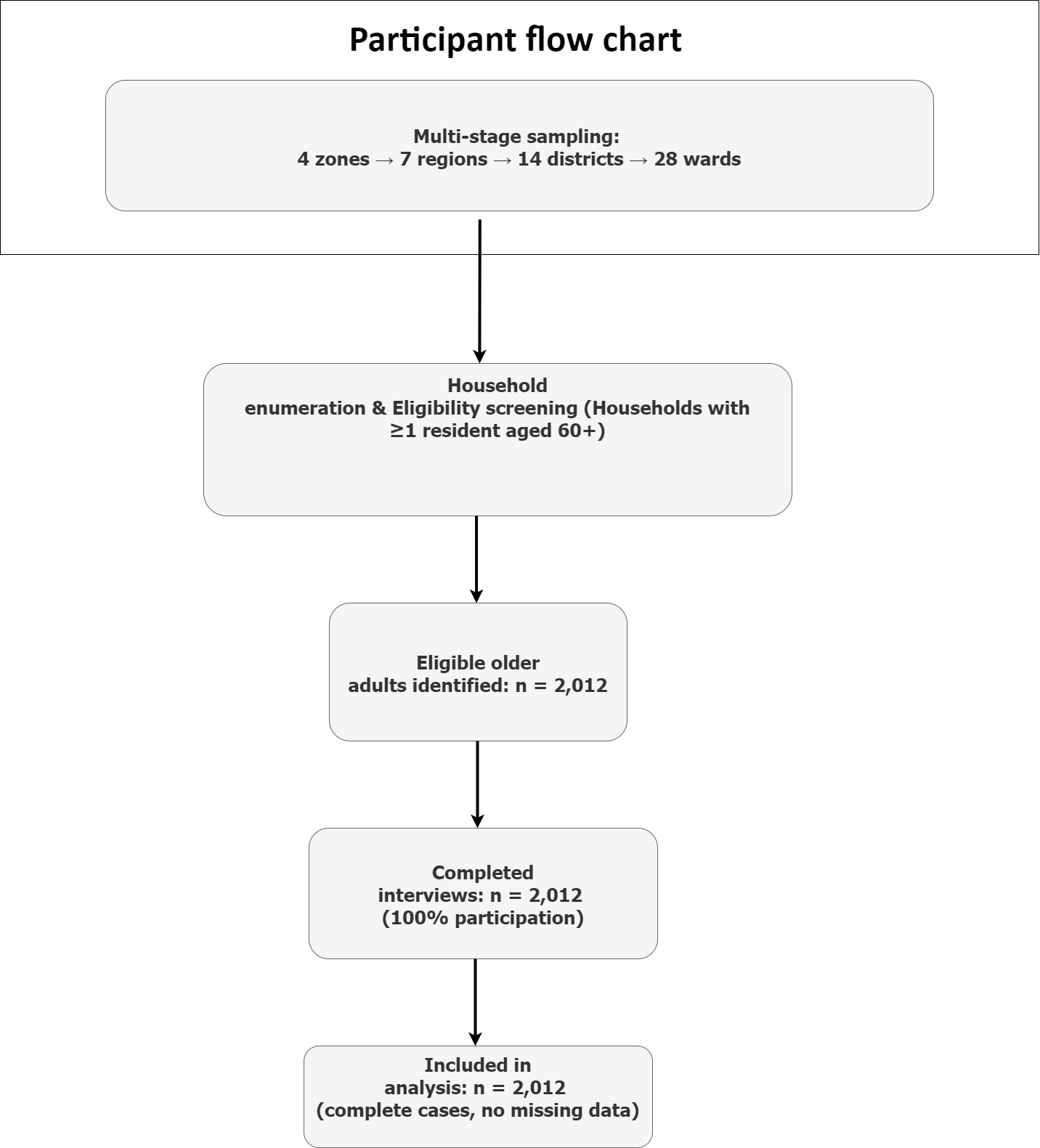


Supplementary Figure 1 Shows participants flows
